# A review and commentary on congenital anomalies of the craniocervical junction

**DOI:** 10.1007/s00247-026-06521-5

**Published:** 2026-02-11

**Authors:** Aaron S McAllister, Eric A Sribnick

**Affiliations:** 1 Department of Radiology, Division of Neuroradiology, Nationwide Children’s Hospital, Columbus, United States; 2 Department of Surgery, Division of Neurosurgery, Nationwide Children’s Hospital, Columbus, United States; 3 Department of Neurosurgery, The Ohio State University, Columbus, United States; 4 Department of Radiology, The Ohio State University, Columbus, United States

**Keywords:** Craniocervical junction, Basilar invagination, Platybasia, Craniometry, Craniocervical junction fusion, Congenital anomalies

## Abstract

This brief review focuses on the imaging of the craniocervical junction with particular attention to linear and angular craniometry. It attempts to answer the question of why there are so many measurements and how they are clinically useful. Congenital anomalies of the craniocervical junction will be discussed with an overview of craniocervical junction embryology and development. Entities affecting the craniocervical junction which will be discussed include platybasia, basilar invagination, atlanto-occipital assimilation, and assorted congenital anomalies.

**Graphical abstract:**

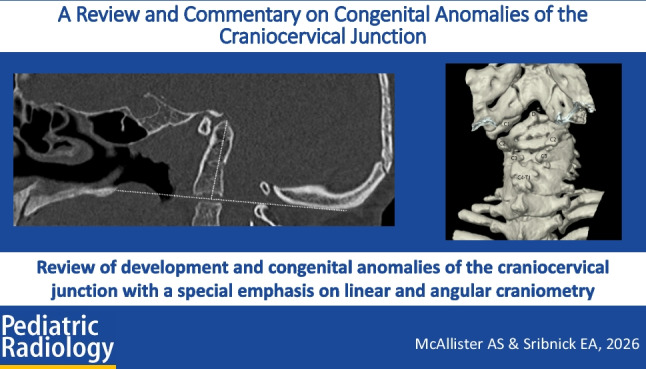

## Introduction

The craniocervical junction is the anatomic region where the cranium joins the cervical spine. It simultaneously provides passage and protection for the brainstem and upper cervical spinal cord while allowing for essential head and neck mobility. Congenital anomalies of the craniocervical junction can manifest as instability, compression, or altered biomechanics, potentially leading to severe neurological sequelae if unrecognized. This review discusses the anatomy, ossification patterns, and embryology of the craniocervical junction, with a particular emphasis on linear and angular craniometry for diagnosing conditions such as platybasia, basilar invagination, and associated anomalies. By addressing the clinical utility and pitfalls of these measurements, we aim to guide radiologists and clinicians in optimizing imaging interpretation.

## Craniocervical junction

**Fig. 1:**
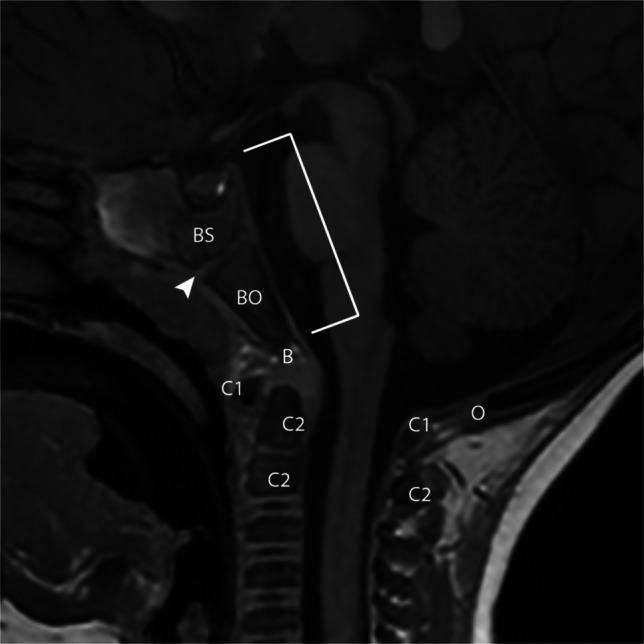
Sagittal T1 image through the skull base in a 9-year-old male. Basisphenoid (*BS*), basiocciput (*BO*), spheno-occipital synchondrosis (*arrowhead*), basion (*B*), opisthion (*O*), visualized components of the C1 vertebral body/atlas (*C1*), visualized components of the C2 vertebral body/axis (*C2*)

The craniocervical junction is comprised of the occipital bone, the atlas (C1), and the axis (C2) with the accompanying stabilizing ligaments. The basilar part of the occipital bone is known as the basiocciput and together with the basisphenoid and the intervening spheno-occipital synchondrosis comprises the clivus. The clivus forms the anterior border of the posterior fossa. The basion is the midline point of the anterior foramen magnum (the tip of the midline basiocciput), while the opisthion is the midline point of the posterior margin of the foramen magnum (Fig. 1).

## Ossification pattern

**Fig. 2:**
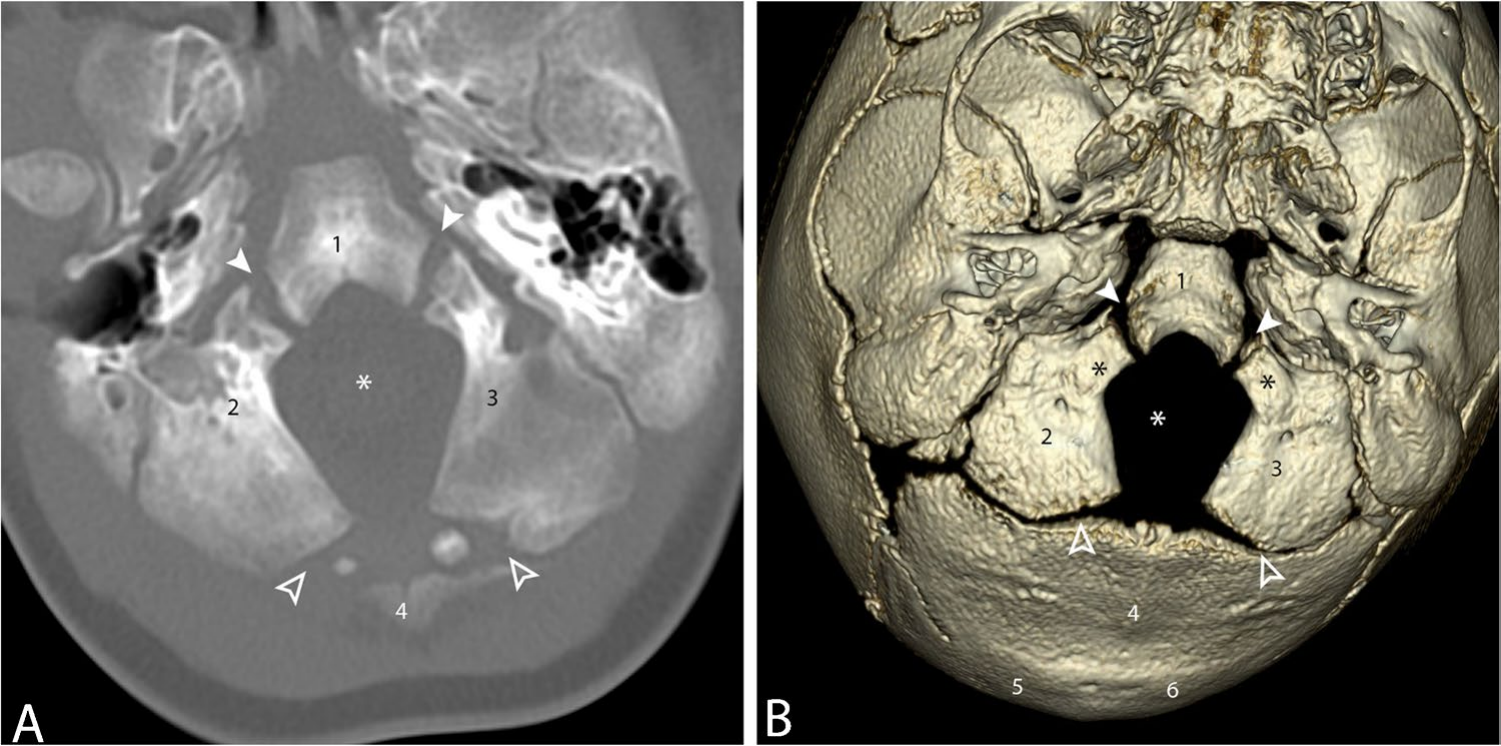
**A** Axial CT through the occipital bone in a 15-month-old male. **B** Inferior view of a 3-D reformation of the occipital bone. There are six ossification centers of the occipital bone. Three develop by enchondral ossification and three by membranous ossification. The three enchondral ossification centers (labeled with *black numerals*) are basiocciput (*1*), the caudal aspect of the clivus; right (*2*) and left (*3*) exoccipital bones, which include the occipital condyles (*black asterisks*). The three membranous ossification centers (labeled with *white numerals*) are supraoccipital (*4*), of which the opisthion is a part, and right (*5*) and left (*6*) interparietal. Note the foramen magnum, denoted by the *white asterisk*, is circumscribed completely by components of the occipital bone. The *white arrowheads* denote the ventral intraoccipital synchondroses and the *hollow arrowheads* denote the posterior intraoccipital synchondroses

There are six ossification centers of the occipital bone: three enchondral ossification centers and three membranous ossification centers. Note that the foramen magnum is completely surrounded by the occipital bone. The clivus/basiocciput forms the anterior margin, the paired exoccipital bones (which include the occipital condyles) form the lateral margins, and the squamosal occipital bone forms the posterior margin. There are two anterior and two posterior intra-occipital synchondroses (Fig. 2) [1].

**Fig. 3:**
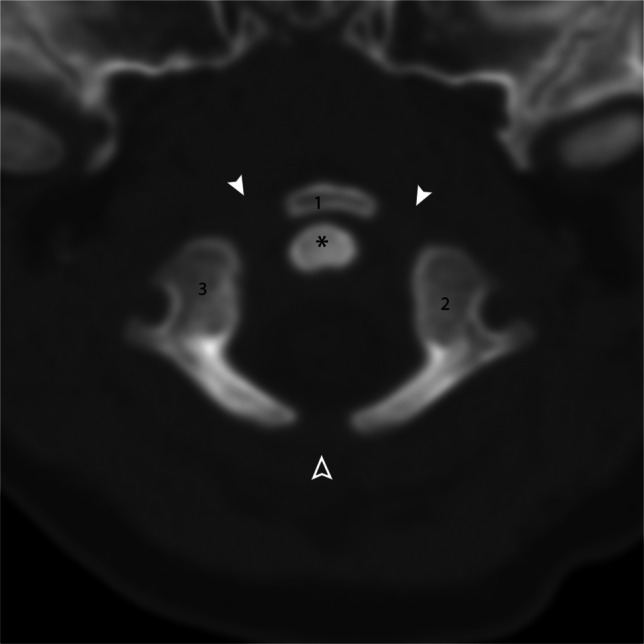
Axial CT of the C1 vertebral body of a 5-month-old male. C1 has three ossification centers: anterior arch (*1*) and paired neural arches (*2* and *3*). Three synchondroses, the paired neurocentral synchondroses (*arrowheads*) and the posterior synchondrosis (*hollow arrowhead*), are present

C1 generally has three ossification centers and three synchondroses; however, there is some anatomic variability in the ossification of C1. Some individuals have no discrete anterior ossification center; the ossification extends medially from the neural arches; most have a single ossification center; some have a bipartite ossification center; and a small minority have multiple and/or irregular ossification centers. C2 has six ossification centers: two comprise the body of the dens, two neural arches, one centrum, and one tip of the dens [2–4] (Fig. 3).

**Fig. 4:**
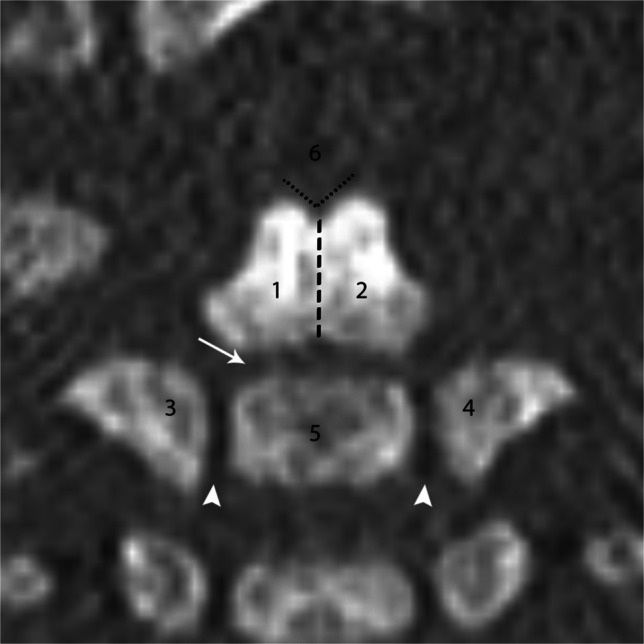
Coronal CT of the C2 vertebral body in a 1-month-old male. C2 has six ossification centers. Two make up the dens (*1* and *2*), two neural arches (*3* and *4*), the centrum (*5*), and the tip of the dens (*6*), which is not yet ossified. There are six synchondroses. The mid-dens synchondrosis (*dashed line*), posterior neural arch (not visualized), subdental (*arrow*), two neurocentral (*arrowheads*), and apicodental (*dotted* *line*), which has a characteristic *v shape*

C2 has six synchondroses. The mid-dens synchondrosis fuses at the seventh fetal month, the posterior neural arch synchondrosis generally fuses between 2 years and 3 years, and the subdental synchondrosis fuses at 3 years to 6 years but is visible until age 11 to 12 as a scar. The neurocentral synchondrosis fuses at 3 years to 6 years old, and the apicodental synchondrosis fuses at 10 years of age, which is the last synchondrosis of C2 to fuse [3, 4] (Fig. 4).

## Biomechanics

The craniocervical junction is a collection of several high mobility joints. According to the in-vitro kinematic study using adult cadaveric material, approximately 50% of flexion of the neck occurs at the occiput-C1 joint, and 60% of neck rotation occurs at the C1-C2 joint [5]. Variations in osseous structures alter biomechanics and range of motion. For example, a higher, narrower, more erect dens increases range of motion. Flatter facets also increase range of motion, and predictive models of how bone morphometry influence range of motion exist [5].

## Embryology

**Fig. 5:**
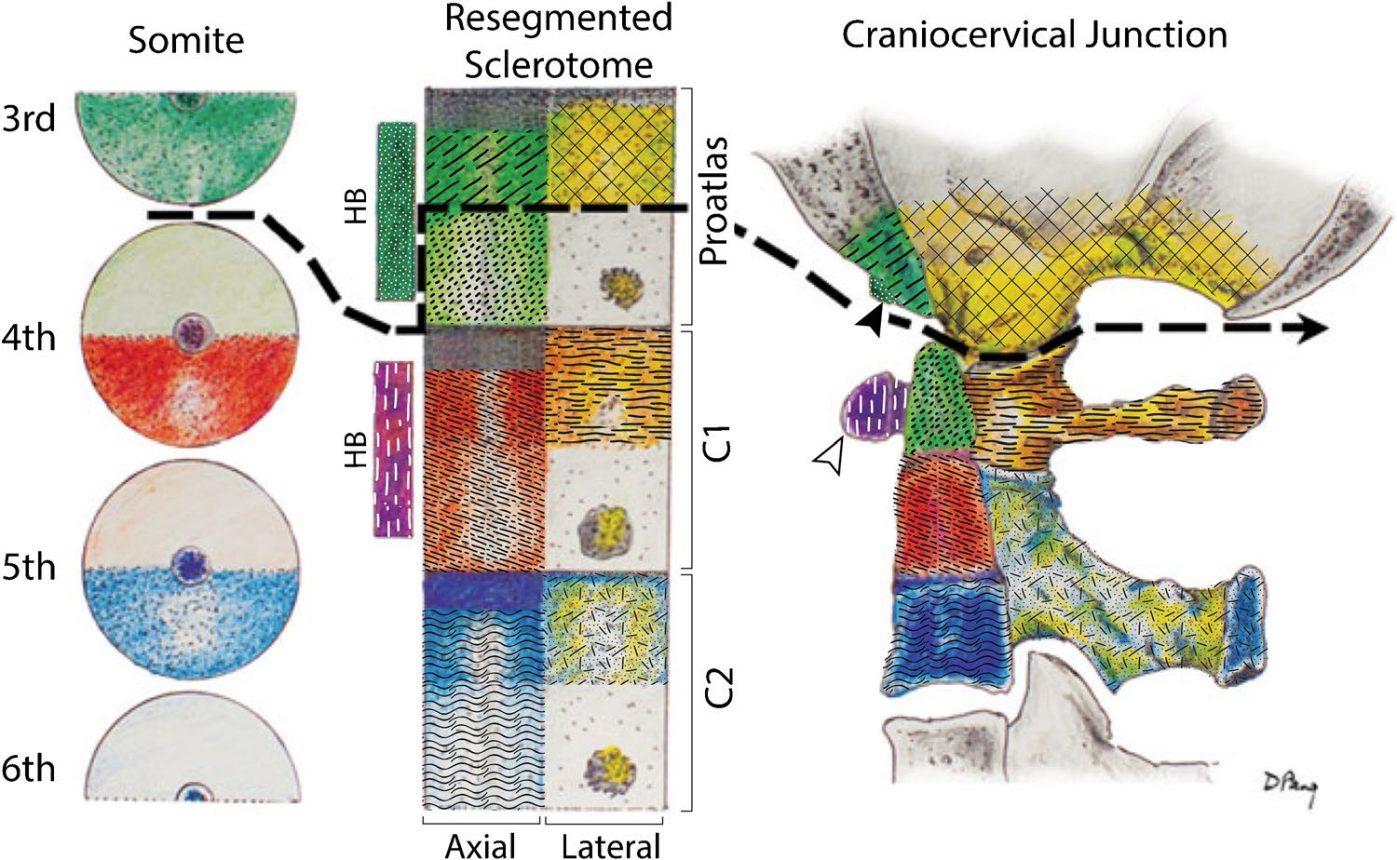
Embryologic development of the craniocervical junction. Somites form in the fourth fetal week and segment into sclerotomes. Sclerotomes differentiate into the craniocervical junction by the sixth fetal week, with ossification beginning the seventh. The craniocervical junction is formed by a cleavage plane through the proatlas sclerotome (*dashed black line*). The C2 vertebral body is formed by contributions from three sclerotomes. The hypochordal bow (*HB*) of the proatlas forms the clival tubercle (*black arrowhead*), while the hypochordal bow of the C1 sclerotome forms the anterior arch of the atlas (*hollow arrowhead*). (Modified from Pang and Thompson 2011. Modifications were made primarily for potential viewing in black and white) [6]

During embryological development, the craniocervical junction is formed from the fourth occipital and the first three cervical somites beginning in the fourth fetal week. The somites reorganize into dermatomes, myotomes, and sclerotomes. The proatlas, C1, and C2 sclerotomes form the craniocervical junction. The proatlas has an axial dense zone that forms the basiocciput, an axial loose zone that forms the apical segment of the dens, a lateral dense zone that forms the exoccipital bones including the occipital condyles, and a hypochordal bow that forms the clival tubercle. A hypochordal bow is a ridge of tissue along the ventral notochord (hence the name). The C1 sclerotome has an axial dense zone and an axial loose zone that together form the basal segment of the dens, a lateral dense zone that forms the lateral masses and posterior arch of C1, and a hypochordal bow that forms the anterior arch of C1. The C2 sclerotome has an axial dense zone and an axial loose zone that together form the body of C2 and a lateral dense zone that forms the posterior arch of C2. A cleavage plane transects the proatlas sclerotome giving rise to the atlanto-occipital joint. It is also notable that the C2 vertebral body receives contributions from three different sclerotomes. The cartilaginous template for the craniocervical junction is complete by the sixth fetal week. Ossification begins in the seventh fetal week [6, 7]. The paper by Pang and Thompson is a great paper for further reading [6] (Fig. 5).

## Craniometry

Before we begin our review of craniocervical junction craniometry, a few words of caution regarding craniometry of the craniocervical junction. It has been said that nothing is more dangerous than a radiologist with a ruler, and an engineering maxim states, the number of methods to measure something is inversely proportional to the suitability of any one of them. Both of these are true with regard to the craniocervical junction craniometry. To that end, many of these angles have similar but not identical landmarks. Each has their specific range of normal and specific use cases. Craniometric ranges of normal are determined by measuring a normative population and selecting a cutoff value based on two standard deviations from the mean or 95% confidence intervals [8–10]. Those patients who fall outside of the normal range are not necessarily symptomatic or pathologic. Some of the most common craniometric measurements, their landmarks, threshold values, and commentary are included in Table 1.

**Table 1:** Angular and linear craniometry names, landmarks, and thresholds

**Craniometry table**				
**Name**	**Landmarks**	**Threshold values and commentary**	**Figure**	**Reference**
McRae Line	From basion to opisthion	Basilar invagination if dens crosses the line	Figure 6	[11]
Chamberlain Line	From tip of hard palate to the opisthion	Basilar invagination if the dens extends >3 mm above	Figure 6	[12]
McGregor Line	From tip of the hard palate to the inferior-most aspect of the occipital bone, which may be the inferior convexity and may lie caudal to the opisthion	Basilar invagination if the dens extends >4.5 mm above	Figure 6	[9, 13, 14]
Wackenheim Line	Along dorsal clivus	Should traverse posterior third of the dens; basilar invagination if odontoid is above	Figure 6	[14]
Bull’s Angle	Angle between the hard palate-opisthion line, and the plane of the atlas	Basilar invagination if >13°	Figure 7	[15]
Height Index of Klaus	Vertical distance measured from the tip of the dens to the tuberculum sella-internal occipital protuberance line	Normal 40–41 mm; Basilar invagination if <30 mm	Figure 7	[16, 17]
Odontoid Tip to Palate-Internal Occipital Protuberance Distance	Vertical distance measured from the tip of the odontoid to the hard palate-internal occipital protuberance line	Abnormal if <9 mm (suggests basilar invagination)	Figure 7	[18]
Digastric Line (Coronal)	Connecting the bilateral digastric grooves	Basilar invagination if <11 mm above the dens	Figure 8	[13]
Bimastoid Line (Coronal)	Connecting the inferior aspects of the bilateral mastoids	Basilar invagination if dens >10 mm above	Figure 8	[13]
Atlanto-Occipital Joint Axis Angle (Coronal)	Between the planes of the atlanto-occipital joints	Condylar hypoplasia if >127° (also elevated in platybasia and basilar invagination)	Figure 8	[19]
Boogaard’s Angle	Measured between dorsal clivus and the basion-opisthion line	Platybasia if >136°	Figure 9	[20]
Basal Angle of Boogaard	Nasion to midpoint of the sella; midpoint of the sella to basion	Platybasia if >143°	Figure 9	[8]
Modified Basal Angle of Boogaard	Floor of the anterior cranial fossa/ethmoid plate to dorsum sellae; dorsum sellae to basion	Platybasia if >125° (typically 14% smaller than the standard Basal Angle of Boogaard)	Figure 9	[8]
Welcher Basal Angle	nasion to tuberculum sellae; tuberculum sellae to basion	Normal 124–142°; platybasia if >145°	Figure 9	[21]
Atlanto-Dental Interval	Distance measured from the posterior cortex of the anterior arch of C1 to the anterior dens cortex	Atlanto-axial instability if >4–6 mm on lateral radiography in neutral position or >3 mm change between flexion and extension	Figure 9	[22]
Space Available for the Cord	Distance measured from the posterior cortex of the dens to the anterior cortex of the posterior arch of C1	Atlanto-axial instability if ≤14 mm (static) or >5 mm change between flexion and extension (dynamic)	Figure 9	[22, 23]
Clivo-Axial Angle	Measured between the dorsal clivus and the dorsal dens	Normal 145–165° (neutral); pathologic if <135° (note up to 30° difference between flexion and extension)	Figure 10	[24]
Clivo-Dens Angle	Measured between mid-dens long axis and the mid-clivus long axis	Basilar invagination if <125° (neutral)	Figure 10	[25]
Clivo-Palatal Angle	Measured between dorsal dens and hard palate	Basilar invagination if <53.5°	Figure 10	[26]

**Fig. 6:**
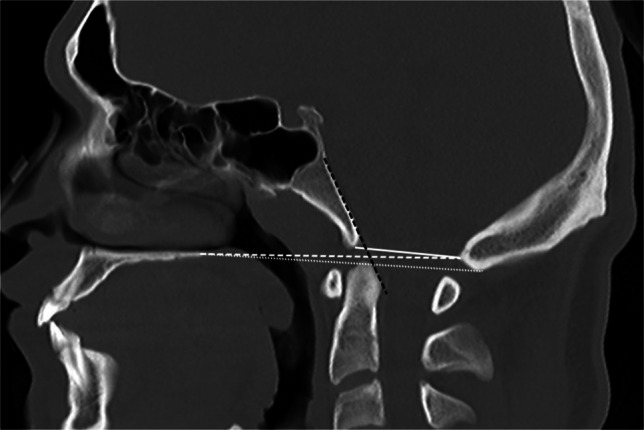
Sagittal CT in a 9-year-old male with a normal craniocervical junction for illustration of craniometric lines commonly used for basilar invagination. The McRae line (*white solid line*) is drawn from the basion to the opisthion. The Chamberlain line (*white dashed line*) is drawn from the hard palate to the opisthion. The McGregor line (*white dotted line*) is drawn from the hard palate to the most caudal aspect of the occipital bone curve. The Wackenheim line is drawn along the dorsal clivus and should traverse the posterior third of the dens (*dashed black line*)

**Fig. 7:**
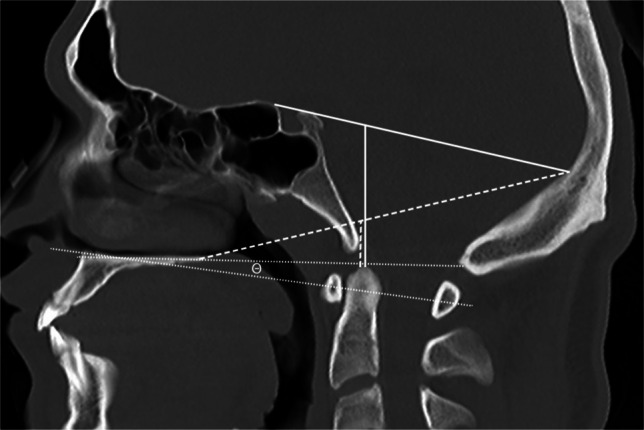
Sagittal CT in a 9-year-old male with a normal craniocervical junction for illustration of craniometric lines commonly used for basilar invagination. Height index of Klaus (*solid vertical white line*) is a length measurement taken from the tip of the dens to a line connecting the tuberculum sellae and the internal occipital protuberance (*horizontal oblique solid white line*). Odontoid tip to palate-internal occipital protuberance distance (*vertical dashed white line*) is a vertical measurement of length from the tip of the dens to a line connecting the hard palate and the internal occipital protuberance (*oblique dashed white line*). Bull’s angle (*theta*) is an angle measured between two lines: the first from the hard palate to the opisthion and the second, a line bisecting both the anterior and posterior arches of the C1 vertebral body (*dotted white lines*)

**Fig. 8:**
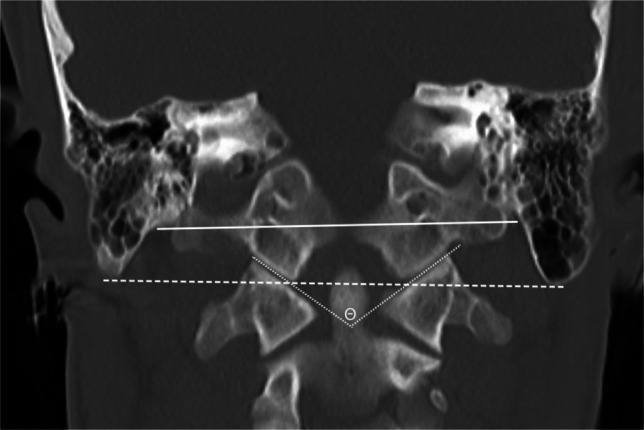
Coronal CT in a 9-year-old male with normal craniocervical junction for illustration of craniometric lines. Digastric line (*solid white line*) is drawn connecting the bilateral digastric grooves. Bimastoid line (*dashed white line*) connects the bilateral most caudal aspect of the mastoid bone. Atlanto-occipital joint axis angle (*white Greek letter theta*) is an angle measured between two lines drawn along the bilateral atlanto-occipital joint (*dotted white lines*)

**Fig. 9:**
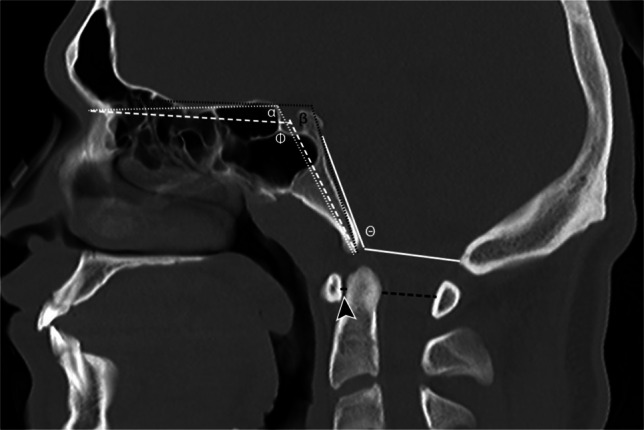
Sagittal CT in a 9-year-old male with normal craniocervical junction for illustration of craniometric lines. Boogaard’s angle (*theta*) is an angle between a line drawn along the dorsal clivus and a line connecting the basion to the opisthion (*solid white lines*). Basal angle of Boogaard (*phi*) is an angle between a line drawn from the nasion to the center of the sella turcica and a second line drawn from the center of the sella turcica to the basion (*dashed white lines*). Modified basal angle of Boogaard (*beta*) is an angle drawn between the line drawn along the ethmoid plate to the tip of the dorsum sella and a second line drawn from the dorsum sella to the basion (*dotted black lines*). Welcher basal angle (*alpha*) is an angle measured between a line drawn from the nasion to the tuberculum sellae and a second line drawn from the tuberculum sellae to the basion (*dotted white lines*). The atlanto-dental interval is a linear measurement taken from the dorsal cortex of the anterior arch of C1 to the ventral cortex of the dens (*black line* and *arrowhead*). The space available for cord is a linear measurement taken from the dorsal cortex of the dens to the ventral cortex of the dorsal arch of C1 (*dashed black line*)

**Fig. 10:**
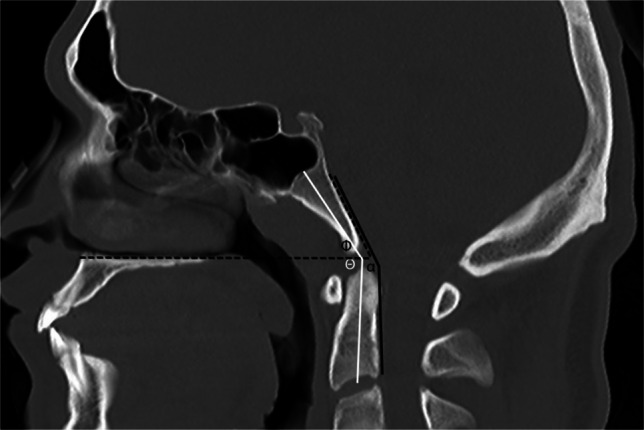
Sagittal CT in a 9-year-old male with normal craniocervical junction for illustration of craniometric lines. Clivo-axial angle (*alpha*) is measured between a line drawn along the dorsal clivus and a second line along the dorsal cortex of C2 (*solid black lines*). Clivo-dens angle (*theta*) is measured between a line longitudinally bisecting the basiocciput and a second line longitudinally bisecting the C2 vertebral body (*solid white lines*). The clivo-palatal angle (*phi*) is measured between a line drawn along the hard palate and a second line drawn along the dorsal clivus (*dashed black lines*)

Why are there so many lines to assess the craniocervical junction? First, landmarks need to be visible in the field of view of the study to be used. Not all landmarks of the craniocervical junction are visible on all study types. For example, the anterior skull base is often excluded on CTs of the cervical spine, while portions of the dens and hard palate are excluded on CTs of the brain. Second, the various measurement techniques were created and validated for specific imaging modalities. For example, the basal angle of Boogaard was originally described for use with plain films. Original landmarks were chosen for optimal visualization for plain films. The modified basal angle of Boogaard was an attempt to update the angle for use on CT and MRI with the choice of landmarks optimized for these modalities. Third, some choice landmarks may not be visible in a specific patient, either because they are not yet ossified or because they have been surgically removed such as opisthion removal as part of Chiari decompression. Landmarks may also be affected by the malformation that is being evaluated and subsequently abnormal in location or morphology. Fourth, the lines measure different components of the same disease. Fifth, abnormalities of the craniocervical junction are rarely isolated. Somites contribute to multiple structures in a complex fashion. Furthermore, if one abnormality is present, altered biomechanics and dynamic load can result in remodeling and altered morphology of the remaining structures.

Given the large number of craniometric methods for use at the craniocervical junction, mistakes are common. Pay close attention to the specific landmarks and normal range used for the specific angle or measurement of choice, noting that many have similar names, and various permutations and normal ranges of values have evolved over time. For example, McGregor introduced his line utilizing 6 mm for females and 7 mm for males [9]. After the introduction of CT and MRI, these values were modified to 4.5 mm for either sex [13, 14].

There is some ambiguity when real patients deviate from the carefully chosen template cases shown in the literature. The posterior borders of the clivus and C2 are rarely straight. Where is the posterior border of a retroflexed dens or dorsally scalloped clivus? Where should the sella or floor of the anterior cranial fossa be drawn in cases of hyper pneumatization of the sphenoid? These questions illustrate some of the variance in these measures in clinical practice.

Skull base craniometry can be helpful, but remember they are most sensitive in the most obvious cases when they are least needed. They are least sensitive when the radiologist is less certain about the diagnosis and most likely to reach for the protractor. Symptoms do not always correlate to the measured values.

## Platybasia

**Fig. 11:**
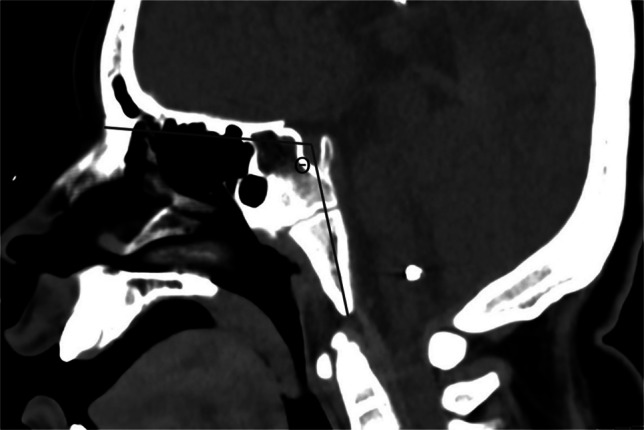
Sagittal CT of the brain in a 10-year-old male. Basilar kyphosis, also known as vertical dens, is when the basal angle is smaller than normative data (the converse of platybasia when the angle is larger than normative data). Basilar kyphosis is of no clinical significance

**Fig. 12:**
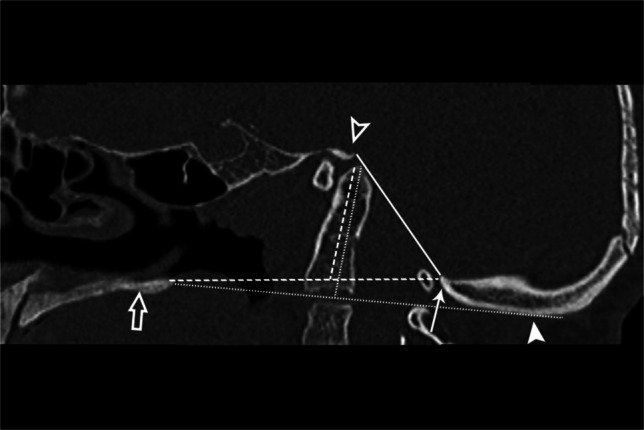
Sagittal CT of the skull base in a 17-year-old female with clear platybasia and basilar invagination. Note that a strict interpretation of the McRae line (*solid line*), drawn from the basion (*hollow arrowhead*) to the opisthion (*arrow*), would not diagnose basilar invagination as the dens does not cross the line. However, the Chamberlain line (*horizontal dashed line*), drawn from the hard palate (*hollow arrow*) to the opisthion, does diagnose basilar invagination as the dens (*vertical dashed line*) extends more than 3 mm above. The McGregor line (*horizontal dotted* *line*), drawn from the hard palate to the lowest portion of the occipital bone (*arrowhead*), also diagnoses basilar invagination as the dens extends more than 4.5 mm above (*vertical dotted line*). Note that superior displacement of the opisthion, which may result in false negatives with the Chamberlain and McRae lines, is accounted for by the McGregor line’s use of the lowest portion of the occipital bone as the landmark

The etymology of the word platybasia is of Greek origin (“platy” is flat and “basia” is base) for flat skull base. In humans, the skull base angle allows upright posture with forward gaze. Isolated platybasia is not clinically important. Boogaard described one of the first basal angles 100 years ago for use on plain films. Landmarks for the basal angle of Boogaard are the nasion to the center of the sella and from the center of the sella to the basion. Platybasia is present if the angle is greater than 143° with normal measurements ranging from 125° to 143°. Alternatively, there is basilar kyphosis if this angle measures less than 125°. Koeningsberg adapted the basal angle of Boogaard for use with CT and MRI, calling it the “modified basal angle of Boogaard.” It is an angle measured between lines drawn along the floor of the anterior cranial fossa/ethmoid plate to the tip of the dorsum sellae and a second line from the tip of the dorsum sellae to the basion. It is a different angle than the basal angle of Boogaard and averages 14% smaller than the standard basal angle of Boogaard. Platybasia is present in kids if the basal angle of Boogaard is greater than 125° (greater than 127° in adults) [8]. Normal basal angle can be seen in Fig. 9, basilar kyphosis in Fig. 11, and platybasia in Fig. 12.

## Basilar invagination

**Fig. 13:**
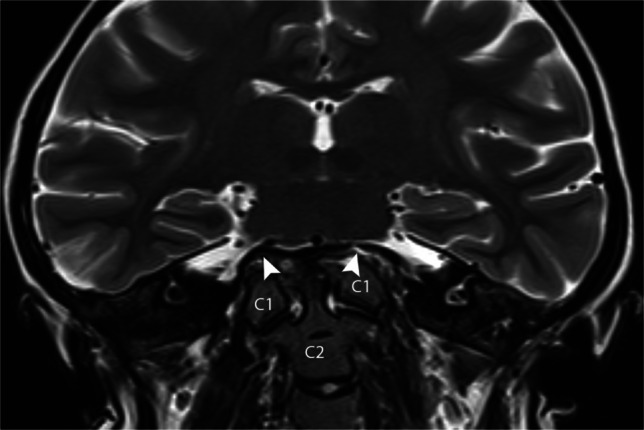
Coronal T2 examination of the brain in a 17-year-old female with basilar invagination. Superior displacement of the upper cervical spine (*C1* and *C2*) with bowing and superior displacement of the skull base (*arrowheads*)

**Fig. 14:**
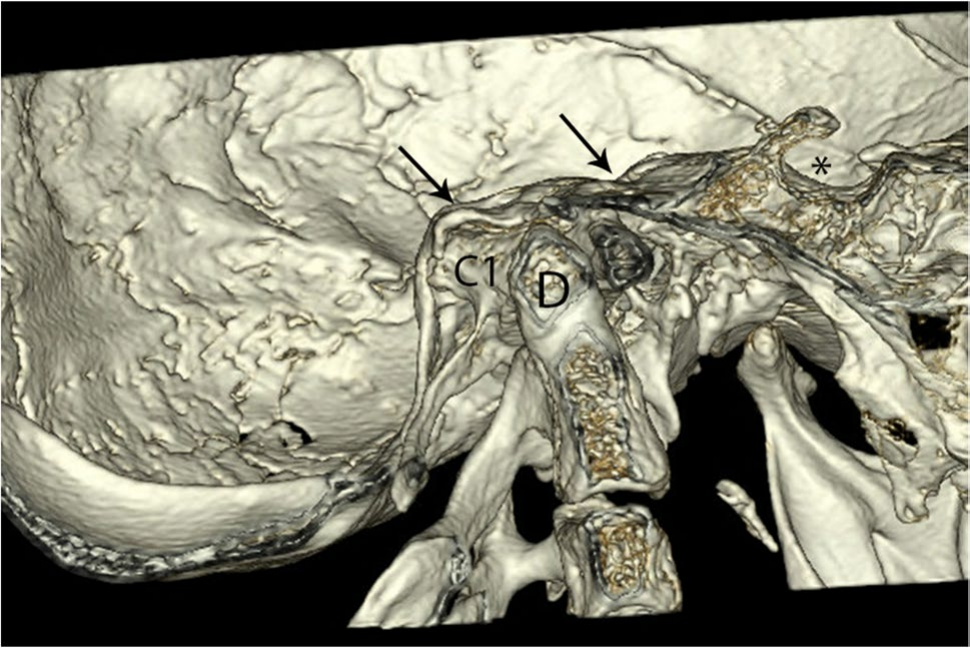
3-D reconstruction of the skull base in a 17-year-old with osteogenesis imperfecta. Note horizontal orientation of the clivus (*arrows*) compatible with platybasia, and superior displacement of the dens (*D*) and atlas (*C*1) compatible with basilar invagination. The sella is denoted by the *asterisk*

Basilar invagination was described by Chamberlain in 1939 as “It is as though the weight of the head has caused the ears to approach the shoulders while the cervical spine, refusing to be shortened, has pushed the floor of the posterior fossa upward into the brain space” [12] (Fig. 13, Fig. 14). Chamberlain used basilar invagination interchangeably with platybasia, but they are two different entities, but commonly cooccur. There are some nuances with regard to terminology. Basilar invagination is congenital. Basilar impression is acquired or secondary to a disease of bone. Basilar settling or cranial settling is secondary to inflammation and resultant joint destruction (e.g., in patients with rheumatoid arthritis). In practice, basilar invagination is used as a general descriptive term while interpreting cases and the other terms, basilar impression or basilar settling, when additional information to justify further classification becomes available. Approximately 30% of basilar invaginations have associated Chiari type 1 malformation in some series, presumed secondary to the space-occupying nature of the craniocervical junction in the posterior fossa [27].

The three most commonly used craniometric lines to diagnose basilar invagination are the McRae, Chamberlain, and McGregor lines. Why do we have three of them? The answer is an interesting illustration of the reasons for the proliferation of craniometric parameters. In many cases, it does not matter which of these lines is used as basilar invagination criteria are met for all three. However, in cases such as that in Fig. 12 with type 2 basilar invagination or high riding dens, a strict interpretation of the McRae line fails to diagnose basilar invagination as the dens does not cross McRae’s line. While Chamberlain’s line that uses the palate, a structure not derived from the proatlas sclerotome, as the landmark, diagnoses basilar invagination as the dens is clearly 3 mm above the Chamberlain line. McGregor’s line can be beneficial in cases with superior migration or upturning of the opisthion as the landmark for McGregor’s line is the most inferior aspect of the curvature of the squamosal occipital bone. The Chamberlain line and the McGregor line are similar in a “normal” skull; however, as the opisthion migrates superiorly with basilar invagination, the most caudal aspect of the occipital bone does not, providing a more reliable measure of invagination. Basilar invagination is present if the tip of the dens is greater than 4.5 mm above the McGregor line [9].

## Occipital bone

**Fig. 15:**
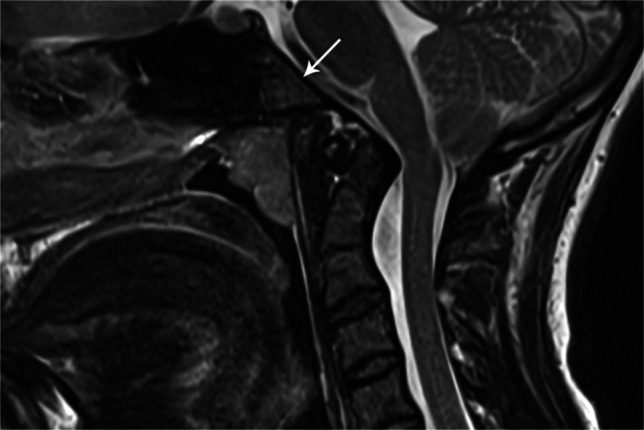
Sagittal T2 image of cervical spine in a 13-year-old female demonstrates a basioccipital hypoplasia (*arrow*)

Basioccipital hypoplasia is a small and/or short basiocciput. Age-based nomograms for basioccipital length are available [28]. Basioccipital hypoplasia may variably contribute to basilar invagination. Concomitant occipital condylar hypoplasia may be present [19] (Fig. 15).

**Fig. 16:**
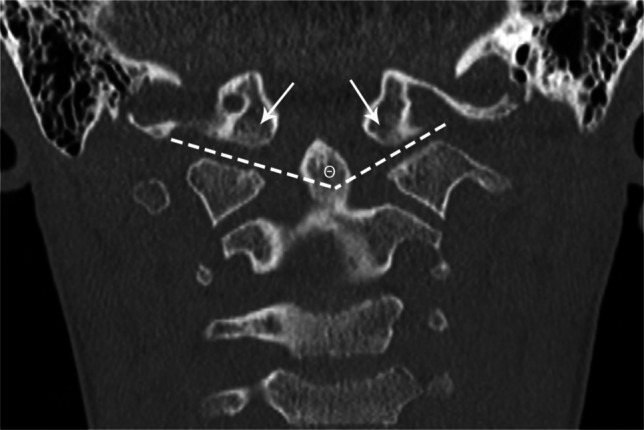
Coronal bone window CT in an 11-year-old female demonstrates abnormally small occipital condyles (*arrows*) compatible with condylar hypoplasia. The atlanto-occipital joint axis angle (*Greek letter theta*), the angle between lines drawn along the atlanto-occipital joints (*dashed lines*), suggests condylar hypoplasia if >127°

Condylar hypoplasia denotes small occipital condyles. The atlanto-occipital joint axis angle, measured as the angle between the right and left atlanto-occipital joints, is larger than 127° in cases of condylar hypoplasia and often in basilar invagination [29] (Fig. 16).

**Fig. 17:**
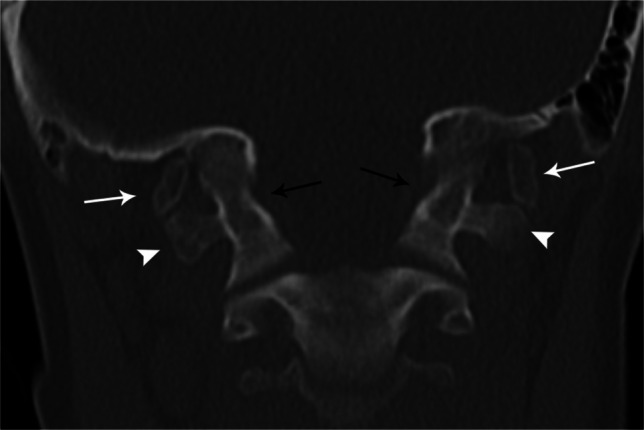
Coronal bone window CT of the spine in a 15-year-old male with bilateral unfused paracondylar processes (*arrows*) with pseudoarticulation with the transverse processes of C1 (*arrowheads*). Note concomitant atlanto-occipital assimilation (*black arrows*)

A paracondylar process is an osseous process arising from the occipital bone lateral to the occipital condyle that may articulate or even fuse with the transverse process of C1. It may result in symptoms such as headache, neck pain, and limited range of motion due to fusion or mass effect (Fig. 17).

**Fig. 18:**
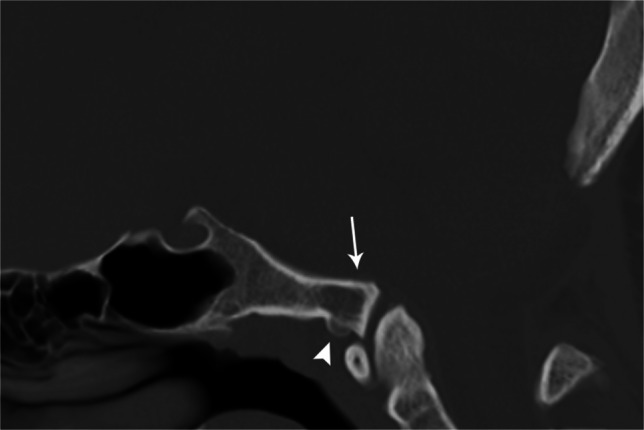
Bone window sagittal CT of the brain in a 13-year-old female demonstrates abnormal widening of the dens with an articular surface for the dens. In some cases, condylus tertius may project inferiorly from the midline basiocciput and may articulate with the anterior arch of C1 and/or the dens. Note the prominent clival tubercle (*arrowhead*), a remnant of the hypochordal bow of the proatlas

Condylus tertius (also known as third occipital condyle) is an enlargement of, or an osseous process arising from, the midline basiocciput with an articular facet for the dens or the anterior arch of C1 [29, 30]. It is postulated to arise from hypertrophy of the ventral tubercle, a remnant of the hypochordal bow, or an accessory ossification center within the cruciate ligament [30] (Fig. 18).

**Fig. 19:**
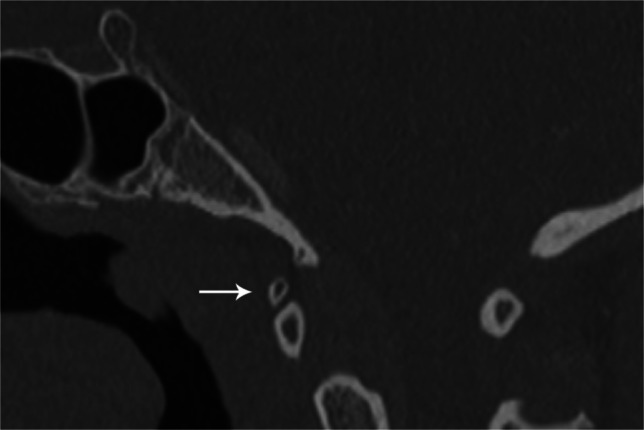
Sagittal CT of the skull base in an 11-year-old female. Accessory ossicle (*arrow*) anterior to the anterior arch of C1. Possibly a remnant of the hypochordal bow. Accessory ossicles can occur in various locations about the craniocervical junction

Accessory ossicles are secondary ossification centers that fail to fuse to the adjacent bone. They are typically small, ovoid, and well corticated. Believed to be primarily congenital in origin, trauma has been suggested as a causative agent for the lack of fusion in some cases. Although more common in the feet and hands, they may occur rarely at the craniocervical junction. Ossicles of the nuchal ligament, accessory ossicles of the atlas, and persistent ossiculum terminale are most common. They are typically asymptomatic incidental findings with the primary diagnostic dilemma distinguishing them from fractures [31] (Fig. 19).

**Fig. 20:**
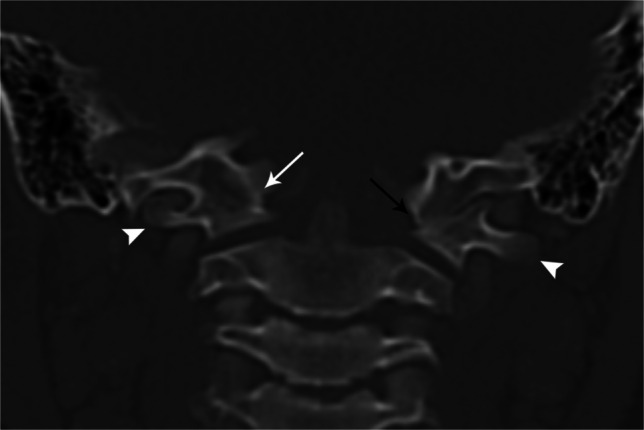
Coronal CT of the neck in a 14-year-old male demonstrates complete atlanto-occipital assimilation on the right (*white arrow*) and incomplete atlanto-occipital assimilation on the left (*black arrow*). The presence of the transverse processes of C1 (*arrowheads*) helps distinguish assimilation from C1 aplasia

Atlanto-occipital assimilation is the abnormal partial or complete fusion of the atlas with the occiput. The prevalence is 0.75–3.75% [32]. Fusion can occur anywhere along the C1 ring including the lateral masses of C1 to the occipital condyles, or the anterior arch of C1 to the basion. The presence or absence of the transverse processes of C1 can be helpful for distinguishing atlanto-occipital assimilation, when the transverse processes are present, from atlas aplasia, when they are absent. As the atlanto-occipital joint is responsible for 50% of the flexion of the head and neck, assimilation alters biomechanics resulting in compensatory hypermobility at the atlanto-axial joint, and subsequent instability over time (Fig. 20).

## Atlas

**Fig. 21:**
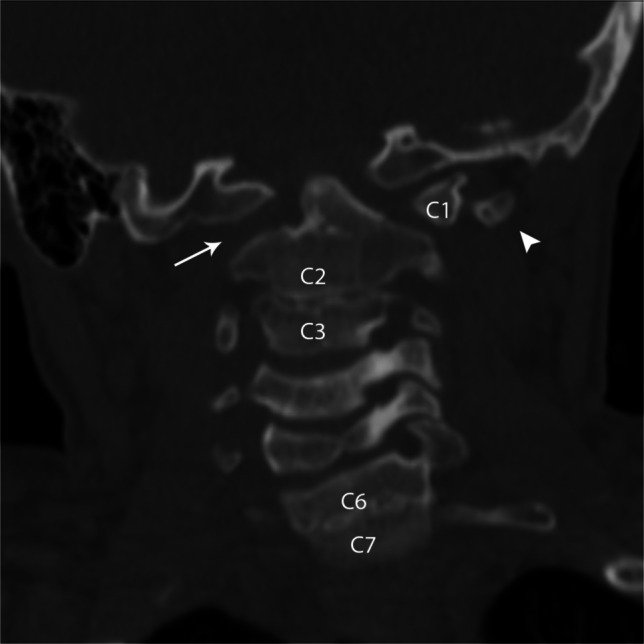
Coronal CT of the cervical spine in an 11-year-old female. Aplastic right C1 (*arrow*) with normal left C1 (*C1*) with normal left C1 transverse process (*arrowhead*). When C1 is aplastic, the transverse process is absent. Multiple segmentation anomalies of the cervical spine including C2 and C3 as well as C6 and C7

**Fig. 22:**
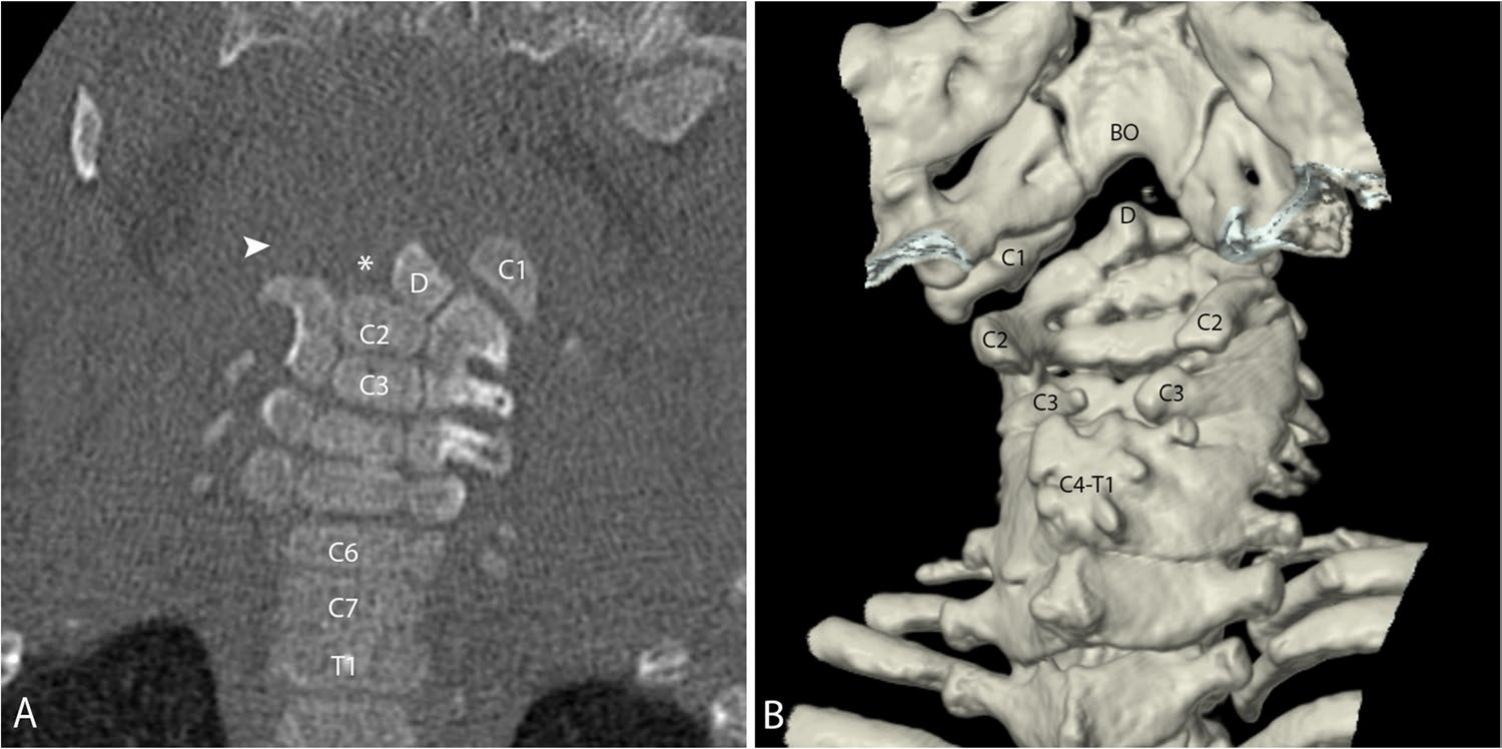
Coronal CT of the brain in a 4-year-old female with Treacher-Collins syndrome. Hypoplasia of the posterior arch of C1 results in a shortened AP dimension of the C1 ring with spinal canal stenosis (*double arrow*). Note the abnormal anterior position of the posterior arch of C1 (*arrowhead*) compared to the posterior arch of C2 (*hollow arrowhead*). As in this case, craniocervical junction findings are often “edge of film” findings on brain imaging

The most common abnormalities of C1 are arch defects/clefts, which may involve any location of the arch. Failure of fusion of the posterior elements is by far the most common and viewed by many as a normal variant [33]. The most important diagnostic consideration is to distinguish arch defects from fractures and look for associated anomalies. The defects may range from a thin cleft to complete aplasia. C1 aplasia can be partial or complete, unilateral or bilateral. Unilateral C1 aplasia is related to the paired sclerotomes from which the vertebral body arises. If only one sclerotome is disrupted, unilateral aplasia results, while bilateral aplasia requires disruption of bilateral sclerotomes (Fig. 21 and Fig. 22).

**Fig. 23:**
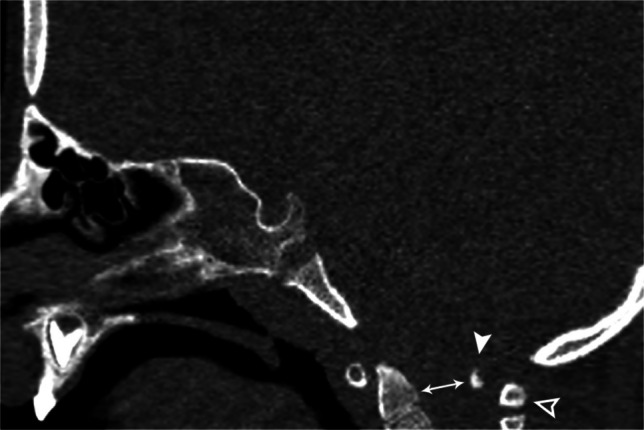
Coronal T1 in a 13-year-old with unilateral atlanto-axial fusion. Note fusion of the right lateral mass of C1 (*asterisk*) to the lateral mass of C2 (*arrowhead*). The presence of the C1 transverse process (*arrow*) can be helpful in distinguishing aplasia from fusion. The left lateral masses of C1 (*C1*) and C2 (*C2*) have a more normal configuration. The occipital condyles (*OC*) and dens (*D*) are labeled for reference

Defects within the C1 ring rarely cause compression as they can act as a congenital laminectomy and generally increase the caliber of the spinal canal, particularly when large [34]. However, a C1 ring with a small internal diameter can result in spinal stenosis, and in severe cases, myelomalacia and resultant neurological symptoms, manifested on imaging by a short internal diameter with narrowing of the space available for the cord [34]. Treatment is posterior decompression with C1 laminectomy (Fig. 23).

## Axis

Aplasia of the axis can also occur and be partial or complete, unilateral or bilateral, and may be isolated to the dens. It is not uncommon for the aplasia to follow the ossification pattern, with the aplastic components corresponding to one or more of the ossification centers (Fig. 22^22^). Like in the atlas, unilateral defects result from interruption of one of the two paired sclerotomes that give rise to C2, while complete aplasia results from disruption of the bilateral sclerotomes.

**Fig. 24:**
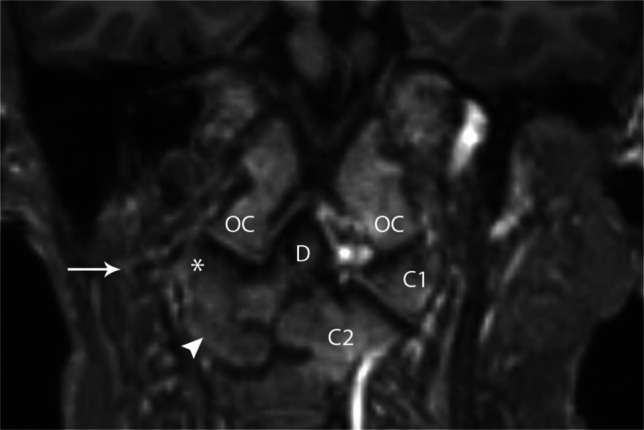
**A** Sagittal T2 MRI and (**B)** sagittal CT in a 13-year-old male demonstrate hypertrophied os odontoideum (*asterisk*) separated from the remainder of C2 by a cleavage plane (*arrowhead*). **B** Subsequent CT demonstrates posterior translation of the os odontoideum (*asterisk*) compatible with instability. Note spinal canal stenosis with cord signal abnormality (*double arrow*)

Incomplete segmentation can also occur with varying degrees of atlanto-axial fusion, which may include fusion of the anterior arch of C1 to the dens, fusion of the posterior elements of C1 and C2, and/or fusion of one or both of the lateral masses (Fig. 24). Similar to atlanto-occipital assimilation, the presence or absence of the transverse processes can be useful in distinguishing aplasia from fusion. As the atlanto-axial joints are responsible for the majority of head/neck rotation, C1/C2 fusion results in altered biomechanics and increased stress on adjacent joints that may degenerate and become symptomatic over time.

## Os odontoideum

**Fig. 25:**
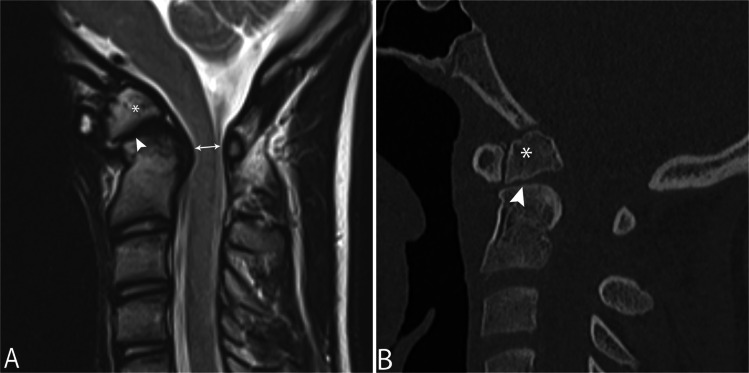
Sagittal T1 of an 18-year-old female. Mild clival dysplasia (*hollow arrowhead*). Basilar invagination with the dens (*white line*) extends >3 mm above Chamberlain line (*dotted line*) combined with mild retroflexion of the dens resulting in mass effect on the ventral medulla. Multiple segmentation anomalies of the spine are also present C2/C3 (*white asterisk*) and C6/C7 (*black asterisk*). The patient had longstanding neck pain and bilateral hand and feet numbness and paresthesia that were unresponsive to bracing. She was treated with anterior decompression and posterior fusion with decreased crowding at the craniocervical junction and resolution of her symptoms

Os odontoideum is a corticated free osseous fragment in the location of the dens. The etiology is uncertain and may be congenital, post traumatic, or related to vascular insufficiency, or a combination of all three [35]. Os odontoideum may be asymptomatic and diagnosed incidentally, or may present symptomatically. Symptoms may be unmasked by minor trauma, increasing the diagnostic dilemma of fracture versus os odontoideum. Corticated margins and commonly observed concomitant C1 anterior arch hyperplasia can be used to distinguish os odontoideum from odontoid fracture. Os odontoideum can result in instability and/or cervical cord myelomalacia. Spinal canal narrowing and/or abnormal cord signal injury may be present. It is treated with atlanto-axial fusion (Fig. 25).

## Klippel-Feil syndrome

The term Klippel-Feil syndrome is an eponym that simply refers to the fusion of two or more cervical vertebrae (Fig. 22).

## Treatment

While many abnormalities of the craniocervical junction are congenital in nature, they develop, progress, and can remodel with child maturation. Sometimes clinical significance is not due to the malformation per se, but the effects of that malformation in critical areas result in symptoms such as neurologic compromise, instability, mobility limitations, and CSF flow alterations. Treatment is primarily based on clinical symptoms with radiological findings as support. Treatment options include physical therapy and bracing, with limited success, or surgery. Surgery can include posterior decompression, anterior decompression, and/or fusion. Fusion is gaining support as the leading treatment.

Neurological symptoms are primarily bulbar (i.e., affect muscles controlled by the lower cranial nerves). This can include findings such as dysphagia, dysarthria, loss of gag reflex, facial weakness, breathing issues, speech changes, and drooling. The etiology of clinical symptoms in craniocervical junction abnormalities is believed to be due to ventral compression on the brainstem, distraction/stretching of the dorsal brainstem, stenosis of the craniocervical junction, lateral compression, or altered biomechanics.

**Fig. 26:**
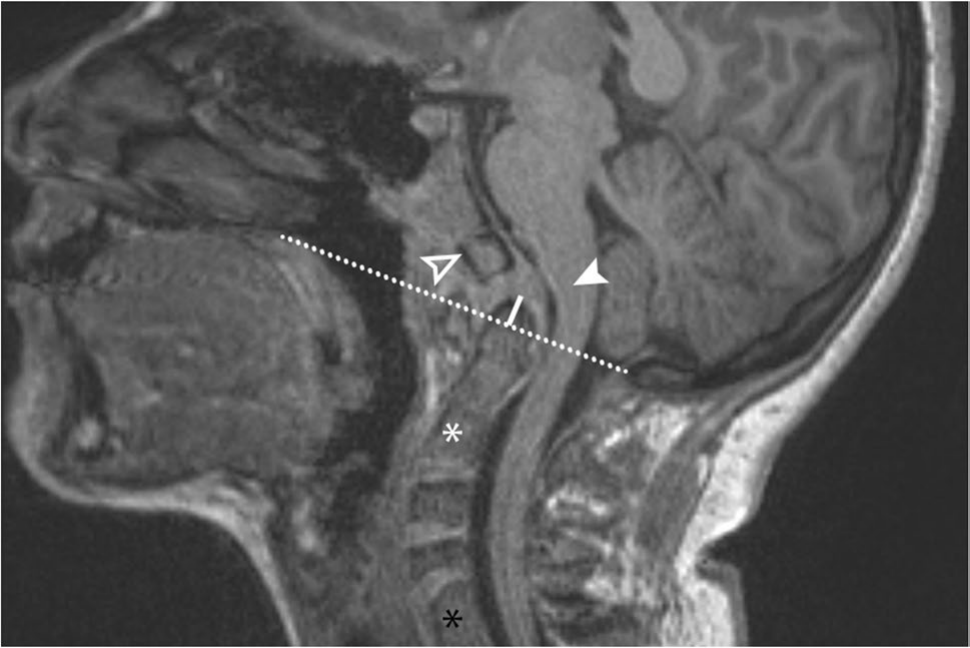
Sagittal T1 of an 18-year-old female. Mild clival dysplasia (hollow arrowhead). Basilar invagination with the dens (white line) extends >3 mm above Chamberlain line (dotted line) combined with mild retroflexion of the dens resulting in mass effect on the ventral medulla. Multiple segmentation anomalies of the spine are also present C2/C3 (white asterisk) and C6/C7 (black asterisk). The patient had longstanding neck pain and bilateral hand and feet numbness and paresthesia that were unresponsive to bracing. She was treated with anterior decompression and posterior fusion with decreased crowding at the craniocervical junction and resolution of her symptoms

**Fig. 27:**
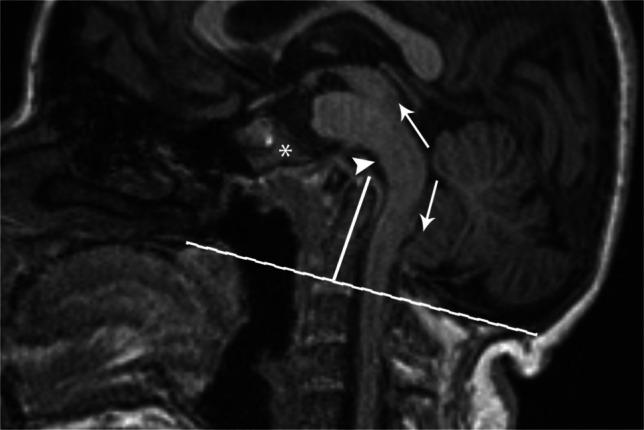
Sagittal T1 MRI in a 17-year-old female. Platybasia, clival hypoplasia (asterisk), and basilar invagination are present. The dens (solid white line) extends >4.5 mm above the McGregor line (dashed line). Imaging appearance does not always correlate with symptoms. Despite the severe imaging appearance, in this specific case, the patient was asymptomatic at the time of imaging. In symptomatic patients, symptoms are primarily bulbar in nature and theorized to be due to one or more of the following mechanisms: direct mass effect on the ventral brainstem (arrowhead), distraction of the dorsal brainstem (arrows), or altered biomechanics at the craniocervical junction.

To illustrate the point that symptoms are more important than imaging appearance, contrast these two cases. The first is an 18-year-old female with long-standing neck pain, bilateral hand and feet numbness, and paresthesias not responsive to bracing. The patient was ultimately treated with anterior decompression and posterior fusion with decreased crowding at the craniocervical junction (Fig. 26), whereas a second case, a 17-year-old female without neurologic symptoms, was treated with serial imaging (i.e., imaging follow-up). Despite the worse appearance by imaging, no surgery was performed due to the lack of bulbar symptoms (Fig. 27).

## Conclusion

In summary, the craniocervical junction represents a complex confluence of joints, ligaments, and bones designed to provide both stability and safety to the underlying vital neurological structures while still allowing for a high degree of mobility. Angular and linear craniometry has its uses in assessing the craniocervical junction, but radiologists must be careful to use the correct landmarks and thresholds. In almost all cases, clinical symptoms, not imaging, drive therapy.

## Data Availability

No datasets were generated or analysed during the current study.
